# Safety Planning for Youth in the Emergency Department Who Have Suicide Risk

**DOI:** 10.1016/j.acepjo.2025.100275

**Published:** 2025-11-06

**Authors:** Ashley A. Foster, Jennifer A. Hoffmann, Kathleen Berg, Tabitha Cheng, Ilene Claudius, Ann M. Dietrich, Gwen Hooley, Samuel H.F. Lam, Joyce Li, Sophia Lin, Donna Mendez, Megan Mroczkowski, Lauren E. Rice, Mohsen Saidinejad, Stephen Sandelich, Genevieve Santillanes, Carmen Sulton, Muhammad Waseem, Theresa Walls

**Affiliations:** 1 Department of Emergency Medicine, University of California, San Francisco, San Francisco, California, USA; 2 Division of Emergency Medicine, Ann & Robert H. Lurie Children’s Hospital of Chicago, Chicago, Illinois, USA; 3 Departments of Pediatrics and Medical Social Sciences, Northwestern University Feinberg School of Medicine, Chicago, Illinois, USA; 4 Division of Emergency Medicine, Dell Medical School, Department of Pediatrics, The University of Texas at Austin, Austin, Texas, USA; 5 Department of Emergency Medicine, Harbor-University of California, Los Angeles, Torrance, California, USA; 6 Division of Pediatric Emergency Medicine, Department of Emergency Medicine, PRISMA Upstate, University of South Carolina School of Medicine, Greenville, South Carolina, USA; 7 Department of Emergency Medicine, Children’s Hospital of Los Angeles, Los Angeles, California, USA; 8 Department of Emergency Medicine, University of California, Los Angeles, Los Angeles, California, USA; 9 Section of Emergency Medicine, Department of Pediatrics, University of Colorado School of Medicine, Children’s Hospital Colorado, Aurora, Colorado, USA; 10 Division of Emergency Medicine, Boston Children’s Hospital, Harvard Medical School, Boston, Massachusetts, USA; 11 Department of Emergency Medicine, Weill Cornell Medicine, New York, New York, USA; 12 Department of Emergency Medicine, The University of Texas Medical Branch Galveston, Galveston, Texas, USA; 13 Department of Child & Adolescent Psychiatry, New York University Grossman School of Medicine, New York, New York, USA; 14 Department of Emergency Medicine, Tufts Medical Center, Tufts University School of Medicine, Boston, Massachusetts, USA; 15 The Lundquist Institute for Biomedical Innovation at Harbor UCLA, Torrance, California, USA; 16 David Geffen School of Medicine at University of California, Los Angeles, Torrance, California, USA; 17 Department of Emergency Medicine, Penn State College of Medicine, Penn State Milton S. Hershey Medical Center, Hershey, Pennsylvania, USA; 18 Department of Emergency Medicine, Los Angeles General Medical Center, Los Angeles, California, USA; 19 Division of Emergency Medicine, Department of Pediatrics, Emory University School of Medicine, Atlanta, Georgia, USA; 20 Department of Emergency Medicine and Pediatrics, Lincoln Medical Center, New York, 10451, USA; 21 Department of Pediatrics, University of Pennsylvania School of Medicine, Philadelphia, Pennsylvania, USA

**Keywords:** suicide, mental health, child, adolescent, risk reduction behavior, emergency medical services

## Abstract

Suicide remains a leading cause of death among US youth. The emergency department (ED) is a critical access point for identifying suicide risk and initiating interventions to reduce that risk. Key strategies include developing individualized safety plans and counseling on reducing access to lethal means. This article reviews the current evidence supporting ED safety planning for youth at risk of suicide and presents a practical framework for its delivery. It also explores strategies to enhance the implementation of safety planning and lethal means counseling, including using clinical pathways, training of staff, optimizing reimbursement, and integrating resources into the electronic medical record system. Finally, the article highlights emerging innovations aimed at improving the reach of safety plan interventions in the ED setting.

## 1 Introduction

Suicide is a leading cause of death among youth aged 10 to 24 years.^1^^,^^2^ Between 2007 and 2021, suicide rates rose by 62%.^1^ In addition, over the last decade, emergency department (ED) visits by youth with suicidality have increased substantially.^3^ In the ED, screening to identify youth at risk for suicide and, when indicated, safety planning, including counseling to reduce access to lethal means, can significantly reduce subsequent suicide attempts.^4^ At present, many EDs do not routinely engage in these risk reduction activities, nor consistently integrate them into discharge education.^5^

A safety plan is a single brief, structured time-limited intervention to support youth at risk of suicide by helping them identify personalized strategies for staying safe during a suicidal crisis. Safety planning is a collaborative effort between a healthcare clinician, the patient, and the family aimed at recognizing early warning signs of a suicide crisis, eliciting personal coping techniques, identifying supportive resources and people, and reducing the availability of means to complete suicide.^6^ A safety plan is action-oriented, equipping youth with clear steps they can take when experiencing suicidal thoughts.

In this paper, we discuss current literature on the safety plan intervention for youth in the ED, provide guidance on how to introduce and deliver safety planning, and explore safety planning implementation strategies and innovations.

## 2 Effectiveness of Safety Planning

Among adults, safety planning is effective in reducing suicidal behavior (combined rate of suicide attempts and suicide deaths) with a relative risk of 0.57 (95% CI, 0.41-0.80)^7^ and is considered a best practice by the Joint Commission and the Suicide Prevention Resource Center.^8^^,^^9^ A landmark randomized controlled trial (RCT) in 2018 demonstrated that safety planning in the ED with adult veteran patients, when combined with structured follow-up calls, decreased suicidal behavior within the subsequent 6 months by approximately half, and doubled the odds of attending at least 1 outpatient mental health visit, when compared with usual care.^6^ When implemented across 8 EDs, a cluster RCT demonstrated that increasing universal suicide risk screening and safety planning among adult patients decreased subsequent suicides and suicide-related health care visits by 43%.^10^

Evaluation of the effectiveness of youth safety planning in the ED has been more limited due to smaller sample sizes and less rigorous study designs.^11^^,^^12^ In 1 RCT of an ED-based pediatric crisis intervention involving families that incorporated safety planning, rates of follow-up care increased compared with usual care (92% versus 76%).^13^ Additionally, among youth in the ED, lower self-efficacy for utilizing key safety planning strategies (eg, calling a crisis hotline, lethal means restriction) was associated with a higher rate of subsequent suicide attempts and return ED visits.^14^ To date, most studies of safety planning in children have included predominantly White females, with none including children aged younger than 10 years.^15^

Despite the known effectiveness of safety planning, gaps exist in implementing safety planning across US EDs. In a survey completed by 513 ED nursing directors, only 15% of EDs routinely provided all recommended safety planning elements.^16^ Provision of each of the 6 individual elements of safety planning ranged from 25% to 79%, with only 2 elements routinely provided more than 50% of the time: lists of professionals or agencies to contact in a crisis (79%) and helping patients to recognize warning signs of suicide (52%). In a large sample of children and adults, patients seen in EDs who reported routinely implementing safety planning were less likely to return to the ED or have an inpatient admission for mental health issues within 30 days.^17^ Additionally, delivery of high-quality, personalized safety plans containing all recommended elements was associated with fewer psychiatric hospitalizations.^18^

## 3 Appropriate Populations to Receive Safety Planning

Patients who are determined to be safe for discharge home after revealing suicidal thoughts or behaviors, self-harm behaviors, or any detected risk on suicide screening should be provided with a safety plan.^19^^,^^20^ Examples of validated screening tools include the Ask Suicide-Screening Questions and Columbia-Suicide Severity Rating Scale (C-SSRS).^21^, ^22^, ^23^ Safety planning should also occur for youth who present with a nonpsychiatric chief concern and have detected risk on suicide screening; this affirms the importance of universal suicide screening, as between 3% and 46% (median 8%) of youth presenting with a nonpsychiatric concern have a positive suicide risk screen.^24^

Depending on local availability of personnel resources, safety plans can be developed by trained nurses, mental health clinicians, ED clinicians, or social workers. They can be developed in person or by telehealth.^25^ Involving caregivers in safety planning is essential, as they can play a key role in monitoring the youth, encouraging use of coping strategies, and reducing access to lethal means.^26^ Clinicians who guide patients in safety planning also must be facile with accessing local resources (eg, community resources, suicide crisis hotlines, see subsection *Professionals or Agencies Who Can Be Contacted During a Crisis* below) and collaborating with outpatient mental health clinicians.^27^ Seventy percent of healthcare clinicians who engage in ED safety planning report a need for more training, underscoring the need for adequate training for staff tasked with this important role.^28^

## 4 Introducing a Safety Plan

Introducing safety planning in the ED before discharge is a crucial task that requires sensitivity, timing, and effective communication.^29^^,^^30^ The healthcare professional’s role is to collaborate with the youth to not only identify safety strategies but also to empower them to take control of their mental health, especially during self-harm or suicide crises.

## 5 Performing Safety Planning

Safety planning is best performed as part of discharge planning, ensuring that the patient and caregiver leave with clear, actionable steps and access to available resources. The safety plan is best introduced when the patient is calm and able to focus. The emphasis of a safety plan is to reduce immediate risk following an ED visit, particularly before the first outpatient follow-up appointment, which may not be available for several weeks.^31^ Normalizing the process by explaining that safety planning is a standard part of care for youth experiencing troubling thoughts or ideas is essential. For example, saying, “We often work with patients to develop a plan for keeping them safe and helping them manage tough moments. I would like to go over a plan like this together,” can be effective. Frame this as a plan that is *created together*. This empowers the patient and promotes a sense of agency. Language tailored to the patient’s emotional state and level of understanding can enhance the safety plan. If the patient appears overwhelmed or disengaged, defer the topic to a more suitable time during the visit. If family or caregivers are present, include them when reasonable, with the patient’s permission. If the patient requests not to include their caregiver, re-discuss confidentiality and its limits.^32^ Help youth understand that when they leave the hospital, their caregiver will need to be able to see how they are doing and help them stay safe.

**Figure 1:**
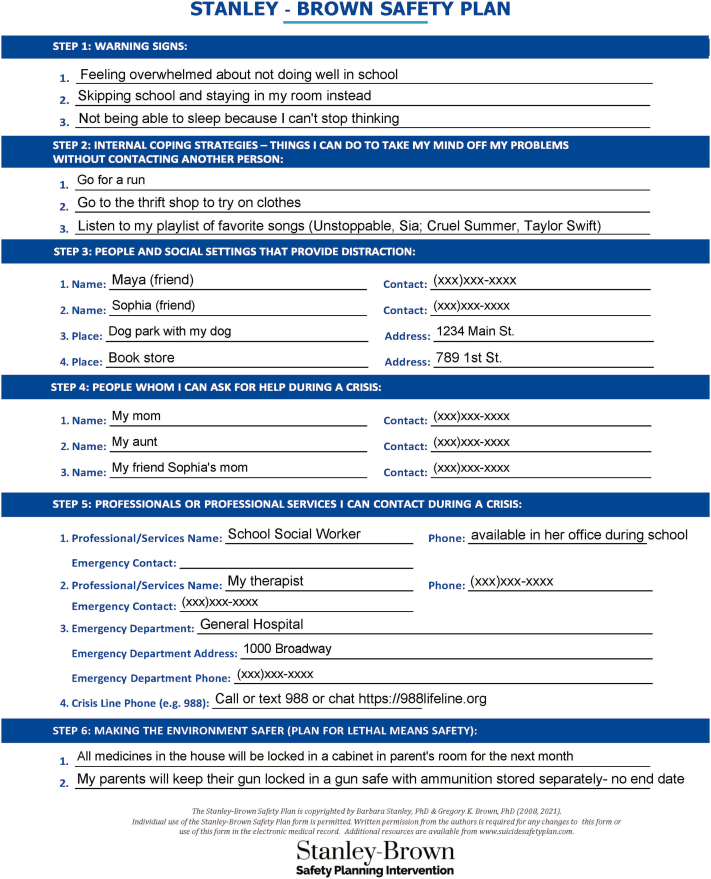
Example of a completed Stanley-Brown safety plan. The figure was adapted from the Stanley-Brown safety plan form and is copyrighted by Barbara Stanley, PhD, and Gregory K. Brown, PhD (2008, 2021). The form and additional resources are available from: https://www.suicidesafetyplan.com.

The goal of safety planning is to reduce risk by developing a preset, personalized, specific, and concrete plan that can be employed in a suicide crisis.^6^ The safety plan includes warning signs, coping strategies such as calming activities and distractions, enlisting supportive people and healthcare clinicians, and lethal means counseling.^6^ The development of a safety plan is a collaborative process that should focus on actions the patient feels comfortable enacting in a crisis. When creating a safety plan, review prior suicidal crises with the patient and discuss the steps in sequential order. The initial steps of the safety plan do not require the patient to reveal that they are in crisis. The Stanley-Brown safety plan is a commonly used framework for conducting safety planning. Forms and training videos are available online to assist in creating a Stanley-Brown safety plan (https://suicidesafetyplan.com/forms/; https://suicidesafetyplan.com/training/). Detailed descriptions of each step of the Stanley-Brown safety plan are outlined below, and an example is shown in Figure 1.

1. *Warning signs*: The first step in the Stanley-Brown safety plan is to recognize warning signs that immediately precede a suicide crisis. These signs may include interpersonal encounters, thoughts, moods, behaviors, or images. Addressing the problem before it fully manifests itself remains one of the most effective ways to avoid a suicide crisis. Warning signs of an impending suicide crisis include feeling depressed, irritable, hopeless, or having suicidal thoughts such as “I can’t do this anymore.” Concerning behaviors may consist of increased social isolation, avoidance behaviors, or increased substance use. Recognition of the warning signs will trigger the use of the safety plan. The more specifically a youth can identify and describe their warning signs, the more effective the safety plan.^33^
2. *Internal coping strategies*: The next step is to determine internal coping strategies tailored to individual youth. Implementing internal coping strategies to cope with suicidal thoughts is key to bolstering self-efficacy to ultimately master suicidal urges. Internal coping strategies mirror distraction techniques described in Dialectical Behavioral Therapy.^34^ Examples of internal coping strategies include listening to music, walking, taking a shower, playing with a pet, reading, creating art, or completing chores.
3. *People and social settings that provide distraction*: The third step in the plan involves reaching out to family or friends without revealing the suicide crisis. Identify specific people, such as friends, whom the youth can contact for distraction. Additional social distractions, which provide a focus away from self-harm or suicidal thoughts, should include safe spaces that are readily available to the youth, such as a nearby park or youth club.^8^
4. *People who can help during a crisis*: The fourth step is to identify trusted adults the youth will be willing to contact and reveal that they are in crisis. These may be caregivers, other family members, teachers, or coaches. Ideally, these adults are aware of the safety plan so they can be optimally prepared to support the youth when they are in crisis.^8^^,^^35^
5. *Professionals or agencies who can be contacted during a crisis*: The fifth step is to identify professionals the youth would be willing to contact in a crisis. This can include primary care and mental health clinicians, as well as school contacts such as counselors. Youth and families can also use the Substance Abuse and Mental Health Services Administration resource, findtreatment.gov, to find treatment for behavioral health symptoms. Identify a place such as an ED, where the youth can access care at any time, as well as a national hotline. The 988 Suicide and Crisis Lifeline provides free 24/7 access to trained individuals.^36^ This hotline is available by calling or texting 9-8-8 or via chat at https://988lifeline.org. Hotlines are also available to support specific populations; for example, the Trevor Project focuses on supporting LGBTQ+ youth (https://www.thetrevorproject.org/).
6. *Making the environment safer*: The last step focuses on making the environment safer. Restriction of lethal means such as firearms, medications, knives, and other sharp objects allows patients time to manage what may be a transient suicide crisis. The safety plan should outline specific steps to be taken to restrict access to lethal means and specify the duration of the restriction. If the youth endorses current suicidal ideation, ask them what means they would consider using in a suicide attempt and document any previous attempts. If the youth denies current suicidal ideation, it is not advised to ask hypothetically what means they would use if they became suicidal. Ask all patients about access to firearms, as there is an increased risk of death by suicide in homes with a firearm, and nearly 90% of suicide acts involving firearms are fatal.^37^^,^^38^ Youth often know the locations of guns even if caregivers believe they are hidden. In 1 study, among caregivers who believed their child did not know the storage location of household guns, 40% of their children reported knowing the location.^39^ After learning about the youth’s access to lethal means, collaborate to identify ways to make the environment safer. Ideally, identify a responsible adult who can appropriately store any lethal means. Options for storing firearms outside the home may include temporary storage with friends/family, gun shops, shooting ranges, and local law enforcement agencies. If firearms are kept in the home, the American Academy of Pediatrics recommends that they be stored locked and unloaded, with the ammunition locked separately.^40^ Safe firearm storage devices include cable gun locks, trigger locks, lock boxes, and safes. In an RCT, distribution of a cable gun lock to caregivers in the ED, when paired with counseling, was more effective than counseling alone in increasing self-reported safe firearm storage 7 days after the ED visit.^41^ Cable gun locks are low-cost and may be acquired free of charge through many local law enforcement agencies and organizations such as Project Childsafe (https://projectchildsafe.org/). However, survey data indicate that caregivers of children in the ED prefer more costly devices such as gun safes.^42^ With respect to medications, another lethal means in youth, access to medications can be decreased by locking both over-the-counter and prescription drugs, limiting the number of accessible pills in each bottle, and discarding medications no longer in use.

## 6 Return Precautions

The transition period from ED to home is a vulnerable period for discharged patients. Youth with behavioral health concerns have a high rate of return to the ED, especially younger children and those with perceived disruptive or self-harm behaviors.^43^^,^^44^ Upon ED discharge, establish clear guidelines on when to return to the ED and delineate a follow-up plan with the youth’s primary care or outpatient mental health clinician. Structured telephone follow-up calls can facilitate the utilization of outpatient resources,^45^ especially for individuals identified as high-risk or those with repeated ED visits. Follow-up phone calls not only serve to connect youth with in-person services but also can connect youth with crisis counselors by phone or text messaging.^46^^,^^47^ Failure to engage with the safety plan, difficulty with outreach to the youth, or with attending follow-up sessions may be warning signs to prompt caregivers to contact their primary care or outpatient mental health professional if they believe their youth’s mental health status is worsening and/or to call 988.^48^

## 7 Implementation Strategies for Safety Planning in the ED

Each ED has unique constraints when caring for suicidal youth and implementing safety planning into clinical workflows. Implementation strategies that may promote the adoption of safety planning in the ED include using clinical pathways, integrating electronic health records (EHRs), staff education, and pursuing reimbursement.

Many US EDs implement suicide screening tools, such as the Ask Suicide-Screening Questions or the C-SSRS Screen Version, which are typically embedded in the electronic health record (EHR) and administered by nurses during triage.^49^ Clinical pathways, such as 1 developed by the National Institute of Mental Health, can standardize the approach taken when youth screen positive, which should include a structured risk assessment, followed by safety planning for youth discharged home.^50^ EHR integration can promote compliance with both screening and safety planning.^51^^,^^52^ Safety planning can be documented in a standardized EHR form or structured templates in the treating team’s notes.^53^ For instance, in 1 ED, minor changes to default EHR note templates were effective in increasing documentation of firearm access from 38% to 82% among youth at risk for suicide.^54^ Documentation of a safety plan in the EHR facilitates ongoing review and adaptation by patients, family members, and outpatient clinicians.

Implementing safety planning in the ED involves educating ED staff, including nurses, clinicians, and social workers, on how to counsel families on limiting access to lethal means, arrange timely follow-up with outpatient mental health professionals, and explore crisis aversion strategies with the patient. Opportunities for training include in-person workshops and virtual training modules.^55^ Examples of free online training courses include Counseling on Access to Lethal Means (https://zerosuicidetraining.edc.org/enrol/index.php?id=20), firearm injury prevention training developed by the BulletPoints Project (https://continuingeducation.bulletpointsproject.org/courses/preventing-firearm-injury/), and training to create a Stanley-Brown safety plan (https://suicidesafetyplan.com/training/).

The use of billing codes to secure reimbursement for safety planning may facilitate implementation into ED workflows. A Centers for Medicare and Medicaid billing code for safety planning is G0560, which can be billed in 20-minute increments.^56^

## 8 Safety Planning Delivery and Innovations

Technology use is ubiquitous among youth^57^^,^^58^ and can enhance safety planning. Web-based decision aids, text messaging, and mobile apps have emerged as affordable and timely strategies to support safety planning.^59^ They can supplement traditional suicide prevention by encouraging real-time monitoring, personalized coping strategies, and immediate access to resources.^60^

Several digital tools have been developed to help youth and families create customized plans to restrict access to lethal means such as firearms.^61^ They are stand-alone (ie, not integrated into the medical record) and can support intervention escalation. One study used a common education platform to automatically alert healthcare clinicians when adolescents exceed suicidal ideation thresholds, based on the Suicidal Ideation Attributes Scale and the C-SSRS, triggering clinician outreach such as safety checks or appointments.^62^ Continued development of digital interventions that safely and securely transmit symptom severity to healthcare clinicians may be beneficial.

Text-message interventions reduce repeat suicidal episodes through personalized and timely crisis support.^63^ Automated text-based safety planning is a novel strategy that allows individuals to create their safety plans by following text-message prompts. In a focus group of young adults, priorities for text-based safety planning included transparency, confidentiality, data security practices, convenience, and accessibility.^64^ Users valued features such as automatic reminders, assistance with developing coping strategies, and mood tracking.^65^

Although app-based innovations with customizable interfaces are under development, no app is currently available that has robust evidence of effectiveness among youth. Examples of apps that are more commonly used and are known include Beyond Now (Google Play), SafePlan, and SmartCrisis 2.0, which allow personalized self-help aid for safety plan creation, real-time guidance, and interactive coping strategies.^7^ Several of these apps have evidence of feasibility and acceptability among users. Preliminary data in adults demonstrate the 3-month efficacy of Beyond Now in reducing suicidal ideation.^66^ Trials regarding the efficacy of SmartCrisis (adults ≥18 years) and SafePlan (adolescents ≥16 years and adults) are ongoing.^64^^,^^66^, ^67^, ^68^, ^69^ In sum, the simplicity, interactivity, and immediacy of digital interventions can contribute to sustainable engagement with evidence-based interventions among tech-savvy youth. Deployment of digital mental health interventions during or after an ED visit may promote accessible, scalable, and personalized crisis support.

## 9 Conclusion

For youth with identified suicide risk who are discharged from the ED, creating a safety plan and counseling on the reduction of access to lethal means are critical interventions to prevent suicide. To support ED implementation, strategies such as clinical pathways, EHR integration, staff training, and optimizing reimbursement may enhance the adoption and sustainability of evidence-based practices to prevent youth suicide.

## Author Contributions

All authors meet the criteria for authorship as outlined by the International Committee of Medical Journal Editors (ICMJE). Each author made substantial contributions to the conception or design of the work, or the acquisition, analysis, or interpretation of data; participated in drafting the manuscript or revising it critically for important intellectual content; approved the final version to be published; and agreed to be accountable for all aspects of the work, ensuring that questions related to the accuracy or integrity of any part are appropriately investigated and resolved.

## Funding and Support

This manuscript did not receive any specific grant from funding agencies in the public, commercial, or not-for-profit sectors. AAF receives funding outside of the submitted work from the Pediatric Pandemic Network. AAF receives research funding for a different study from Abbott Laboratories. The content presented here is that of the author(s) and does not necessarily represent the official views of, nor an endorsement by HRSA, ASPR, HHS, or the U.S. Government. For more information, visit HRSA.gov. JAH received support from the National Institute of Mental Health of the National Institutes of Health under Award Number K23MH135206-01. The content is solely the responsibility of the authors and does not necessarily represent the official views of the National Institutes of Health.

## Conflict of Interest

All authors have affirmed they have no conflicts of interest to declare.
